# Mitochondria orchestrate proteostatic and metabolic stress responses

**DOI:** 10.15252/embr.201947865

**Published:** 2019-09-18

**Authors:** Claes Andréasson, Martin Ott, Sabrina Büttner

**Affiliations:** ^1^ Department of Molecular Biosciences The Wenner‐Gren Institute Stockholm University Stockholm Sweden; ^2^ Department of Biochemistry and Biophysics Stockholm University Stockholm Sweden; ^3^ Institute of Molecular Biosciences University of Graz Graz Austria

**Keywords:** mitochondria‐to‐nucleus signaling, organellar connectivity, protein folding, proteostasis, stress response, Metabolism, Post-translational Modifications, Proteolysis & Proteomics, Protein Biosynthesis & Quality Control

## Abstract

The eukaryotic cell is morphologically and functionally organized as an interconnected network of organelles that responds to stress and aging. Organelles communicate via dedicated signal transduction pathways and the transfer of information in form of metabolites and energy levels. Recent data suggest that the communication between organellar proteostasis systems is a cornerstone of cellular stress responses in eukaryotic cells. Here, we discuss the integration of proteostasis and energy fluxes in the regulation of cellular stress and aging. We emphasize the molecular architecture of the regulatory transcriptional pathways that both sense and control metabolism and proteostasis. A special focus is placed on mechanistic insights gained from the model organism budding yeast in signaling from mitochondria to the nucleus and how this shapes cellular fitness.

**Glossary**
AAA+: ATPase associated with various cellular activities
ADP: Adenosine diphosphate
ATP: Adenosine triphosphate
cAMP: Cyclic adenosine monophosphate
ER: Endoplasmic reticulum
Hsf1: Heat shock factor 1
HSP: Heat shock protein
HSR: Heat shock response
IPTP: Interorganellar proteostasis transcription program
ISRmt: Integrated mitochondrial stress response
MAGIC: Mitochondria as guardian in cytosol
MHC: Major histocompatibility complex
mitoCPR: Mitochondrial compromised protein import response
mPOS: Mitochondrial precursor over‐accumulation stress
OXPHOS: Oxidative phosphorylation
PKA: Protein kinase A
ROS: Reactive oxygen species
sHSP: Small heat shock proteins
SRP: Signal recognition particle
TORC1: Target of rapamycin complex 1
TOR: Target of rapamycin
UPRam: Unfolded protein response activated by mistargeting of proteins
UPR: Unfolded protein response

## Introduction

Eukaryotic cells comprise cooperating organelles that are interconnected and communicate in a finely tuned system. Disturbances of these connections provoke and modulate disease, including cancer and degenerative disorders 1. Mitochondria are best known as the power plants of the cell that provide a highly efficient pathway to produce ATP via oxidative phosphorylation (OXPHOS). In addition, mitochondria play multiple important roles in cellular physiology, e.g., various biosynthetic pathways are at least partly localized to this organelle and cellular signaling is modulated by mitochondrial release of ROS and calcium. Notably, mitochondria have pivotal roles in cellular decisions whether to die or to survive with the release of pro‐apoptotic factors from the mitochondrial intermembrane space constituting a key event in apoptosis signaling 2, 3. As a consequence of the crucial role of these organelles for the cell, mitochondrial dysfunction is linked to deteriorated physiology with direct effects on cellular stress response, aging, and disease. Thus, exploring how mitochondria are wired in the eukaryotic cell is central to understanding physiology and pathophysiology.

Aging is a process that is controlled by the integrated metabolic and proteostatic systems in the cell. Thereby, metabolic alterations influence the proteostasis system that via feed‐forward connectivity drives the aging program. This raises the possibility that the maintenance of the proteome is a central process downstream of multiple longevity pathways and that misfolded or aggregated proteins accelerate aging 4, 5. Indeed, many of the lifespan controlling pathways influence the proteostasis system, aggregated proteins accumulate during aging and experimentally inducing pathways that fortify the proteostasis system is sufficient to increase lifespan in diverse organisms including yeast, worms, and flies 6. The nature of the proteostasis system with its constant influx of newly translated proteins that have to undergo organellar targeting and folding to avoid off‐pathway misfolding may provide the basis for its rapid decline during aging 7. Misfolded and aggregated proteins will tie up key folding and quality control factors and thus accelerate the decline of proteostasis systems by impairing the folding of newborn proteins. Widespread loss of protein function and the accumulation of numerous species of misfolded and aggregated proteins are processes likely to damage cellular function and the wiring of the metabolic and proteostatic pathways. The interconnectivity and feedback control of organellar sub‐system proteostasis could therefore explain its collapse during aging 1.

Mitochondrial function and the proteostasis system in the cytoplasm and nucleus are intimately linked. For example, mitochondrial dysfunction is frequently caused by defects in the OXPHOS system that consists of the respiratory chain and the ATP synthase 8, 9. Importantly, the majority of the OXPHOS complexes are protein mosaics encoded by both nuclear and mitochondrial DNA. Therefore, assembly of the respiratory chain and the ATPase requires import of many nuclear‐encoded proteins and the coordinated synthesis of a handful of proteins within mitochondria. Thus, the two genetic systems with their respective protein quality control and proteostasis sub‐systems are interconnected to maintain mitochondrial function. Perturbation of the cytosolic proteostasis system has negative consequences for the downstream import of newly synthesized proteins into mitochondria. Conversely, mitochondrial dysfunction may hamper import of proteins from the cytosol and thus burden the cytosolic proteostasis system with the accumulation of mistargeted precursor proteins. This interconnectivity necessitates complex control systems to coordinate not only the proteostasis system but also fundamental metabolism to ensure the function of mitochondria.

Mitochondrial dysfunction has been associated with the opposite outcomes of shortened and prolonged lifespan in different organisms 10, 11. For example, a steady decline in the capacity to synthesize mitochondrially encoded proteins correlates well with human aging 12. In line, rapid accumulation of damaged mitochondrial DNA in a mouse model expressing an error‐prone mitochondrial DNA polymerase provokes premature aging 13, 14. Yet, decreased activities of certain mitochondrial factors instead extend lifespan in many organisms, including yeast 15, 16, 17. The budding yeast *Saccharomyces cerevisiae* represents one of the main model organisms used to study cellular stress and aging. This unicellular eukaryote can undergo two distinct types of aging, commonly used to unravel the processes underlying aging of mitotic and post‐mitotic higher eukaryotic cells, respectively. Replicative aging refers to the number of daughter cells one individual mother cell produces before entering senescence, and chronological aging describes the time cells stay viable in the stationary, post‐mitotic phase 18. One notable difference between the aging of post‐mitotic yeast and animal cells is their energetic state: While chronologically aged yeast cells are nutrient‐starved, long‐lived animal cells such as neurons are not. Nevertheless, aging of yeast and mammalian cells has been shown to impact mitochondrial functionality. Moreover, mitochondrial (dys)function has been shown to determine replicative as well as chronological lifespan.

About three decades ago, loss of mitochondrial DNA was shown to trigger distinct changes in nuclear gene expression in yeast, providing the first evidence for the existence of retrograde signaling from mitochondria to the nucleus 19. Since then, numerous mitochondrial stress regimes have been shown to elicit distinct mitochondria‐to‐nucleus communication. As an outcome, transcriptional reprogramming adapts metabolism and proteostasis in the cytoplasm and mitochondria. Thereby, different signaling routes respond to a plethora of insults, including hampered mitochondrial protein import, mitochondrial protein aggregation, dissipation of mitochondrial transmembrane potential, drop in ATP levels, defective oxidative phosphorylation, loss of mitochondrial DNA, and distinct changes in mitochondrial translation 20. While mitochondria‐to‐nucleus communication exists in organisms ranging from yeast, worms, and flies to mammals, the molecular mechanisms employed to achieve this interorganellar signaling can differ between species. Here, we will review the current knowledge of the cooperation between mitochondrial and cytoplasmic proteostasis and metabolism. The key concept that emerges is that cooperation is achieved by sensing the flow of metabolites and proteins, resulting in remodeling of mitochondrial and nuclear gene expression to shape cellular physiology and aging. We propose that future research efforts should be directed toward understanding the fluxes of unfolded proteins and metabolites within the interconnected proteostatic and metabolic systems.

## Communication between mitochondria and the nucleus at a glance

**Figure 1:**
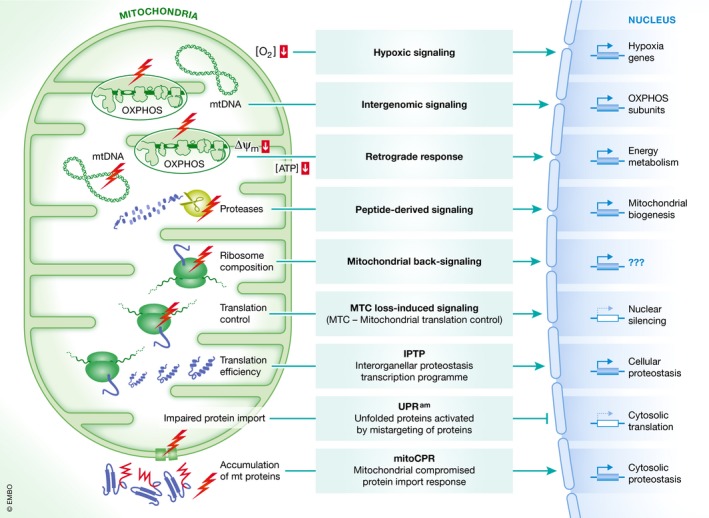
Mitochondria‐to‐nucleus communication pathways Various signaling routes transmit information from mitochondria to the nucleus in response to disturbances in mitochondrial proteostasis or bioenergetics. Distinct pathways are activated upon compromised protein import, accumulation of mitochondrial proteins, alterations in mitochondrial translation, loss of mitochondrial DNA, impaired respiration, and dissipation of the mitochondrial transmembrane potential and trigger transcriptional reprogramming to adapt cellular proteostasis and metabolism. MTC, mitochondrial translation control; IPTP, interorganellar proteostasis transcription program; UPR^am^, unfolded protein response activated by mistargeting of proteins; mitoCPR, mitochondrial compromised protein import response.

Various interorganellar communication routes transmit the mitochondrial energetic and proteostatic states to the nucleus, triggering transcriptional reprogramming to adapt proteostasis systems and metabolism (Fig 1). For example, in response to compromised mitochondrial protein import and thus disturbances of the proteostatic flow between the cytosol and mitochondria, cells downregulate cytosolic translation and simultaneously upregulate proteasomal activity. This pathway, termed “unfolded protein response activated by mistargeting of proteins” (UPR^am^), counteracts the accumulation of mitochondrial proteins in the cytosol 21. Alternatively, the “mitochondrial compromised protein import response” (mitoCPR) is activated upon accumulation of mitochondrial precursor proteins at the mitochondrial surface and within the import channel 22. MitoCPR enables the clearance of accumulating precursors from the mitochondrial import machinery. Those signaling pathways appear to be independent of respiratory capacity and thus mitochondrial bioenergetics and exclusively react to compromised proteostatic flow between the cytosol and mitochondria.

Another mitochondria‐to‐nucleus signaling pathway independent of respiration but dependent on the presence of mitochondrial DNA is “intergenomic signaling”. This coordinates the expression of mitochondrial proteins encoded on the nuclear and mitochondrial DNA to ensure proper OXPHOS complex assembly 23. Moreover, mitochondrial proteases degrade any excess OXPHOS subunits, generating small peptides that subsequently can be released from mitochondria 24, 25. These peptides have been proposed to act as a signal to modulate nuclear gene expression to adapt OXPHOS complex biogenesis to mitochondrial status 26. Details of this *peptide‐derived signaling* and how these peptides are sensed or exert any signaling function remain unclear. Worth noting in this context is that mitochondrially derived peptides have for a long time been known to function as antigens for mouse MHC class I‐b 27, 28. Besides these pathways, distinct alterations in mitochondrial proteostasis, in particular mitochondrial translation, have been shown to elicit transcriptional reprogramming. These communication routes include “mitochondrial back‐signaling”, a pathway that is induced upon lack of a specific mitochondrial ribosomal protein 29. Perhaps related, *loss of mitochondrial translation control‐induced signaling* is induced by the inactivation of mitochondrial translation activators, resulting in altered nuclear gene expression and genome silencing 30. Both of these pathways are independent of mitochondrial respiration. By connecting mitochondrial translation efficiency and cellular proteostasis, we could recently establish that mitochondrial translation efficiency is a key regulator of mitochondrial and cytosolic proteostasis, involving an “interorganellar proteostasis transcription program” (IPTP) 17.

Besides mitochondria‐to‐nucleus communication pathways responding to distinct cues of the interconnected proteostasis systems, additional pathways exist that react to mitochondrial bioenergetics and metabolism. Here, the best characterized pathway is the “retrograde response”, which is induced upon loss of mitochondrial DNA, defects in respiration or dissipation of the mitochondrial transmembrane potential. The retrograde response activates a transcriptional program that adapts cellular metabolism to compromised respiration 31, 32, 33. In response to decreased oxygen levels, another transcriptional pathway termed “hypoxic signaling” is activated that allows cells to cope with the increased production of reactive oxygen and nitrogen species 34.

In sum, a plethora of different signaling routes transmit information from mitochondria to the nucleus to reprogram gene expression and physiology. A common theme among these signaling pathways is that they respond to perturbations of mitochondrial proteostasis and bioenergetics to induce stress‐transcription programs in the nucleus. For most pathways, details of how mitochondrial functionality is monitored and information transferred to specific gene‐regulatory factors are unclear. Likewise, the target genes of the signaling pathways are in many cases not well defined. As a consequence, the extent of overlap and the likely interaction between these signal transduction pathways require further investigation. In light of the intricate connection between mitochondrial integrity and cellular age, most if not all of these pathways are likely to influence stress tolerance and lifespan.

## Cellular proteostasis systems

The proteome is an integrated system that displays robustness toward perturbations of individual components due to redundancy and compensatory mechanisms 1. Yet, every system has its tolerance limits, and a high‐enough stress load will push the proteome over an irreversible limit characterized by widespread protein misfolding and aggregation. Thus, a continuously increasing stress load on the proteome ultimately brings the system to an irreparable state that impedes cellular fitness.

### Fluxes through the proteostasis system

**Figure 2:**
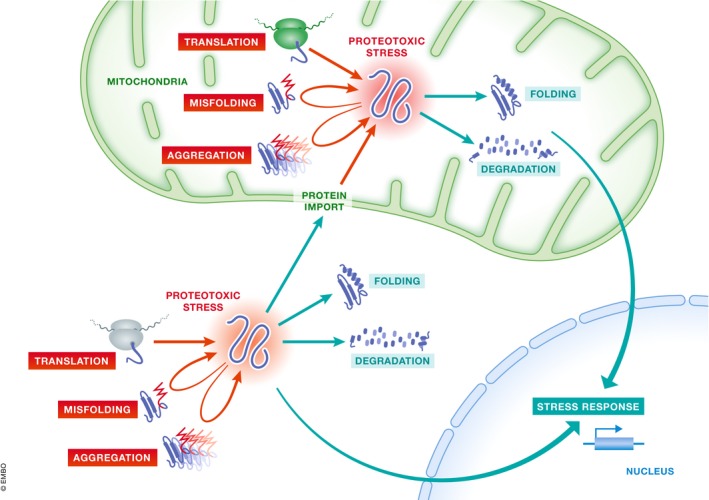
Flow through the proteostasis system The net in‐ and outflow of polypeptides in non‐native conformation determines the state of the proteostasis system. Newly synthesized proteins exiting the ribosome as well as misfolded proteins constitute the major inflow, while organellar import of newly synthesized peptides (e.g., into the ER and mitochondria) and protein folding as well as proteolytic degradation represent the main outflow from the cytosolic proteostasis system.

The state of the proteostasis system is determined by the net in‐ and outflow of potentially toxic polypeptides in non‐native conformations (Fig 2). Proteins exit the ribosomes in unfolded states that transiently associate with ribosome‐associated chaperones 35. As a consequence, newly synthesized proteins constitute the major inflow of substrates in the cytosolic proteostasis system. On the contrary, direct sorting and organellar targeting of newly synthesized proteins provide a substantial outflow. Specifically, a large fraction of the proteins synthesized in the cytosol will be targeted to organelles, mainly the ER and mitochondria, and handled by the organelle‐specific proteostasis system. The other major inflow into the proteostasis system is caused by protein damage and misfolding. These processes belong to normal physiology but are greatly accelerated by stress. Chaperone‐assisted and spontaneous protein folding are responsible for the lion share of the outflow from the cytosolic proteostasis system. Proteins that resist folding are removed from the cell by the ubiquitin–proteasome system and autophagy, two proteolytic pathways that provide an outflow from the proteostasis system by degrading misfolded proteins. Alternatively, they are shunted to insoluble assemblies termed protein aggregates for storage.

Interestingly, organellar and mitochondrial targeting has recently emerged as an important quality control mechanism that aids cytosolic proteostasis. Perhaps most strikingly, cytosolic misfolded proteins have been proposed to be handled via the MAGIC (mitochondria as guardian in cytosol) pathway that imports them into mitochondria for the efficient proteolytic degradation within the organelle 36. On a similar note, the peptidyl‐tRNA hydrolase Vms1 binds to stalled cytosolic ribosomes at the mitochondrial surface and aids the import of these aberrant polypeptides into the organelle for intra‐mitochondrial quality control 37, 38. Organellar proteins that fail to target other organelles such as the ER appear to by default be imported into mitochondria. Specifically, blocking ER‐targeting via SRP inactivation results is extensive mistargeting of secretory proteins to mitochondria 39. The mitochondrial targeting is likely regulated at the level of the ribosomal tunnel exit since inactivation of the nascent polypeptide‐associated complex in *Caenorhabditis elegans* results in widespread import of mitochondrial proteins into the ER 40. In sum, imbalances in any proteostatic sub‐system not only result in depletion of functional proteins but also compromise proteostatic fluxes within other organellar sub‐systems.

Cytosolic and mitochondrial proteostasis are tightly connected, and failure or overloading of either one proteostasis system has severe consequences for cellular fitness with widespread loss of protein and organelle function and extensive protein aggregation. Under normal physiology, the cells battle such dire consequences by functional redundancy and by homeostatic mechanisms that adjust the distinct proteostasis systems to deal with different stress loads. As mentioned above, these compensatory mechanisms include the transcriptional reprogramming via UPR^am^ and mitoCPR. Further, the assembly of the OXPHOS complexes within mitochondria requires the coordination of several processes including nuclear and mitochondrial gene expression, translation in both compartments, mitochondrial import of the newly synthesized proteins in the cytoplasm, and, finally, assembly of the membrane‐embedded complexes 41.

On the regulatory side, signaling routes are in place that ensure a coordinated synthesis of OXPHOS components encoded in the nuclear and mitochondrial DNA 42. Failure of the proteostasis system impacts on aging with consequences for lifespan 1, 7, 43. Presently, it is unclear whether collapse of proteostasis and the concomitant functional decline of the cell during aging occur spontaneously or in a programmed fashion. Conceptually, the proteostasis system may malfunction as a consequence of failure of the regulatory circuits that adjust it to cellular needs or due to overloading of the system by extensive protein misfolding induced by aging. In the first scenario, the supply side is limiting due to regulatory breakdown, while in the latter case, the demand for refolding has increased due to increased protein misfolding. Importantly, in both scenarios, the outcome will be similar: The proteostasis system lacks the capacity to deal with the load of non‐native polypeptides and they will accumulate as proteotoxic species.

### Shunting misfolded proteins to reversible aggregates

Flooding the proteostasis system beyond its capacity shunts the misfolded proteins to insoluble aggregates. Protein aggregation decreases the load on the soluble proteostasis system by shielding problematic hydrophobic peptide segments from soluble proteostasis factors. Aggregation in the cell is a highly orchestrated process. Cells employ stress‐inducible factors that drive aggregation. The most widespread family of such aggregation factors are the small heat shock proteins (sHSP), which are major constituents of aggregates 44, 45. Examples of other, structurally distinct, aggregation factors are the highly stress‐inducible Hook family members Btn2 and Cur1, which promote protein aggregation at peripheral cytosolic and nuclear sites 46. Aggregated proteins will be disentangled and reactivated by dedicated ATP‐fueled chaperones 47. In this way, misfolded proteins are rescued by the chaperones without the need to employ the substantially more energy‐consuming processes of degradation and *de novo* synthesis 48.

Both in the cytosol and in mitochondria, disaggregation relies on generalist and specialized chaperones that remodel the surface to enable extraction of the protein by a disaggregase/unfoldase. The notable players for the generalized chaperones are the Hsp70 and Hsp110 family members and for the specialized chaperones the AAA+ unfoldases such as Hsp104 in the cytosol and Hsp78 within mitochondria 47, 49, 50, 51, 52. This chaperone machinery is not likely to function on the mitochondrially encoded polytopic membrane proteins of the respiratory chain complexes. These highly hydrophobic proteins are cotranslationally inserted into the mitochondrial inner membrane, and aberrant forms that threaten the membrane integrity are instead cleared by turnover 53. Thus, the aggregation mechanisms at play to ensure the functional expression of proteins that originate in the cytosol may differ considerably from the setup for mitochondrially encoded membrane proteins.

Worth noting is that the activity of AAA+ ATPases is not restricted to disaggregation. In mitochondria, they are involved in protein degradation and even biosynthesis. For example, the bacterially derived ClpXP combines the unfoldase activity of the AAA+ ATPase (ClpX) with feeding the disentangled polypeptide directly to a tethered protease (ClpP) 54. Mitochondrial ClpX has an additional protease‐independent role in the biogenesis of 5‐aminolevulinic acid synthase by stimulating the incorporation of the cofactor pyridoxal phosphate 55. These activities are important to hinder the build‐up of aggregated protein.

With progressing age, aggregates accumulate in the cytosol of yeast cells. These aggregates display first as multiple small peripheral spots that eventually increase in size and associate with the ER and the mitochondrial membrane 56. The physiological significance of this membrane localization of the aggregates is not understood, but it is likely that lipid microenvironments on the membrane surface offer platforms for hydrophobic interactions that concentrate the potentially toxic misfolded protein species 57, 58. Conceptually, the age‐dependent accumulation of protein aggregates may be the consequence of (i) an increased load on the proteostasis system resulting in accelerated shunting of misfolded proteins to aggregates, (ii) aggregates with distinct properties that make them resistant to disaggregation, or (iii) a functional decline of the disaggregation machinery with cellular age. As cytosolic translation is downregulated when cells decrease their growth rates during chronological aging 59, an increased load on the proteostasis system via protein biogenesis is unlikely. A more plausible explanation is that ROS derived from increased mitochondrial activity following the diauxic shift damages proteins that in turn burden the proteostasis system 60. Along this line, oxidatively damaged proteins generate aggregates that are more difficult to disentangle. These aggregates might tie up the disaggregation machinery due to stalling of Hsp104 on the chemically modified substrates. Alternatively, these proteins can result in futile cycles of disentanglement followed by instant re‐aggregation due to hampered folding. Still, routes other than ROS are likely to provide immediate communication between mitochondria and the cytosolic disaggregation system. For example, we have observed that a decreased mitochondrial translation output immediately liberates cytosolic disaggregation capacity 17. Finally, the proteostasis system may lose functionality during aging, resulting in the accumulation of protein aggregates. This might result from transcriptional downregulation and also from mechanisms that directly regulate chaperones. For example, Hsp104 is an unusual ATPase that exhibits higher affinity for ADP than ATP, with the consequence that a low energy status, as it occurs during cellular aging, results in ADP inhibition of the disaggregase 61. Other ATP‐dependent chaperones such as Hsp70 are high‐affinity ATP binders and are not predicted to be affected by decreased energy levels. Most probably, several of these mechanisms contribute to the build‐up of aggregated proteins during aging.

## Transcriptional responses to proteostasis stress

The cell monitors the load on the proteostasis system and modifies its gene expression to maintain proteostasis. A burdened proteostasis system decreases chaperone availability and, as a consequence, increases the expression of chaperones to meet the demand. The mechanistically best understood eukaryotic proteostasis gene‐regulatory networks are two ancient pathways, the unfolded protein response (UPR) and the heat shock response (HSR). The UPR senses proteostasis in the ER (UPR^ER^) and conveys the information to the nucleus to regulate a broad set of genes involved in maintaining ER proteostasis 62. In contrast, the HSR reacts to the accumulation of misfolded proteins in the cytoplasm and activates the highly conserved transcription factor Hsf1 that regulates a broad set of proteostasis genes encoding chaperones, co‐chaperones, and aggregation factors. While the UPR and the HSR are controlled by the load on the proteostasis system, pathways regulated by metabolic stress converge on many of the HSR and UPR genes. In yeast, the major metabolic stress pathway involves the kinase PKA, which modulates the activity of the two transcription factors Msn2 and Msn4 (Msn2/4) that bind the promoters of many HSR genes 63. Hence, the transcriptional regulation of the proteostasis system is wired to coordinate the proteostatic and metabolic state. For instance, Hsf1 and Msn2/4 are activated during heat shock by widespread protein misfolding and metabolic perturbation, respectively. The outcome is a broad transcriptional program that impacts the core proteostasis system as well as metabolism.

### Transcriptional changes triggered by compromised mitochondrial protein import

The flow of proteins through the cytosolic and mitochondrial proteostasis systems regulates transcriptional programs. Perhaps the best‐known example is Hsf1 that is regulated in response to the load on the cytosolic proteostasis system by chaperone‐repression titration 64. According to this model that recently has gained critical experimental support 65, Hsp70 chaperones bind Hsf1 and maintain it inactive. An increased load on the proteostasis system results in titration of Hsp70 by unfolded proteins with the consequence that transcriptionally active Hsf1 is released. Conceptually, such cytosolic titration of a chaperone repressor forms the basis of a communication route from mitochondria to the nucleus. Accordingly, impaired mitochondrial import results in Hsp70 titration by the precursor proteins that the chaperone escorts to mitochondria and, as a consequence, activation of Hsf1. Such perturbations might involve proteostasis defects within mitochondria since import is motored by the ATPase activity of mitochondrial Hsp70 chaperones that also expedites general protein folding within the organelle 66, 67, 68. Recent work demonstrates that mitochondrial stress fortifies the cytosolic proteostasis system in an Hsf1‐dependent manner 10. Specifically, mutations weakly perturbing the mitochondrial electron transport chain in *C. elegans* were shown to counteract the age‐related decline of the HSR and cytosolic proteostasis. In *C. elegans*, mitochondrial import efficiency determines the fate of the transcription factor ATFS‐1 in UPR^mt^ 69, 70. While under normal conditions, ATFS‐1 is imported into mitochondria for degradation, it escapes import upon mitochondrial dysfunction. This allows its nuclear accumulation to regulate genes related to organellar proteostasis 71.

In yeast, a recent study showed that overexpression of proteins that rely on a bipartite signal sequence for their import into mitochondria hampers mitochondrial protein import and triggers the induction of nuclear gene expression in the course of mitoCPR 22. Although not the focus of the study, inspection of the 217 genes induced by the overexpression of a bipartite signal sequence‐containing protein reveals that they include the classical Hsf1 target genes, highlighting that mitochondrial protein import indeed is an important determinant for cytosolic proteostasis and Hsf1 regulation. This is in line with previous data showing that Hsf1 upregulates genes required for mitochondrial import 72. Notably, another transcription factor, Pdr3, is responsible for the transcriptional reprogramming during mitoCPR 22. This confirms previous studies demonstrating that mitochondrial stress induced by organic solvent activates Pdr3 via a retrograde signaling pathway from mitochondria to the nucleus 73. Interestingly, the regulation of Pdr3 is known to be reminiscent of the control of Hsf1 and to involve Hsp70 chaperone repression 74.

Collectively, these studies illustrate that mitochondrial energy and proteostasis perturbations cause import defects that result in the accumulation of precursor proteins in the cytosol. This burden on the cytosolic proteostasis system titrates Hsp70 capacity and in response activates chaperone‐repressed Hsf1 and Pdr3. These transcription factors reprogram cellular gene expression to restore proteostasis by upregulating chaperones and factors that remove misfolded proteins from mitochondria. The fluxes of newly synthesized proteins through the cytosol and their import into mitochondria are the critical determinants that underlie the regulatory mechanisms controlling proteostasis.

### Transcriptional changes in response to altered mitochondrial translation

**Figure 3:**
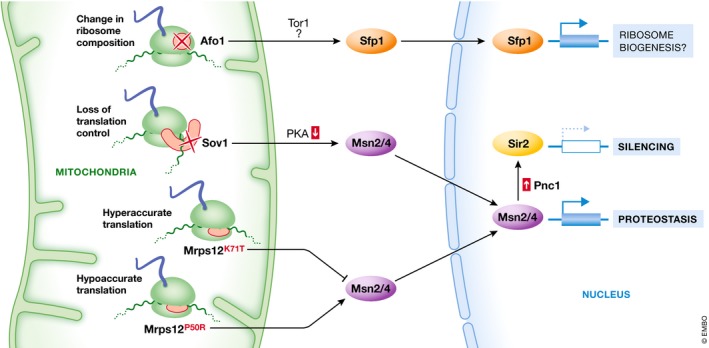
Transcriptional reprogramming in response to alterations of the mitochondrial translation machinery Modifications in mitochondrial translation or mitoribosome composition trigger distinct transcriptional changes that impact aging. The absence of the mitoribosomal subunit Afo1 activates mitochondrial back‐signaling involving the transcription factor Sfp1, an essential regulator of cytosolic translation. The loss of mitochondrial translation control, e.g., in the absence of Sov1, impacts on PKA signaling and the Msn2/4 stress response and activates Sir2‐mediated gene silencing. Mutations that alter the accuracy of mitochondrial translation trigger transcriptional changes via the Msn2/4 stress signaling pathway that affect cellular proteostasis and aging.

Modifying the control or accuracy of mitochondrial translation as well as the absence of distinct mitoribosomal subunits all trigger distinct transcriptional responses that impact aging (Fig 3). Specifically, activation of mitochondrial back‐signaling via deletion of *AFO1*, encoding a mitoribosomal protein, results in resistance to oxidative stress, decreased ROS production, and an extension of replicative lifespan 29. Moreover, the abrogation of mitochondrial translation control via inactivation of translational activators such as Sov1 extends replicative lifespan, however in this case without affecting cellular oxidative stress levels 30. Interestingly, despite that loss of mitochondrial DNA and concomitant respiratory deficiency can result in prolonged replicative lifespan 15, the longevity in these both cases is independent of respiration 29, 30. Instead, deletion of *AFO1* reverses the slow growth of cells lacking mitochondrial DNA, indicating that modifying the mitochondrial translation can suppress the changes provoked by the loss of mitochondrial DNA 29. In cells lacking mitochondrial DNA, mitoribosomes are not assembled due to the absence of ribosomal RNAs, yet a plethora of ribosomal protein subunits are expressed and futilely imported into the organelle. This poses a substantial burden to the mitochondrial and likely also to the cytosolic proteostasis system. Why the lack of a single mitoribosomal protein alleviates such stress is puzzling and could suggest that Afo1 plays a direct regulatory role.

The regulatory circuits involved in prolongation of replicative lifespan upon alterations of mitochondrial translation involve canonical nutrient signaling pathways. The lack of Afo1 results in nuclear translocation of the Tor1‐responsive transcription factor Sfp1, which is a master regulator of ribosome biogenesis and cytosolic translation (Fig 3, top) 29. Downregulation of cytosolic translation via depletion of the ribosomal population or reduced Sfp1 activity are regimes known to prolong lifespan 75. Still, loss of mitochondrial translation control via *SOV1* deletion does not impact cytosolic translation. Instead, deletion of *SOV1* induces gene silencing via the histone deacetylase Sir2 that is essential for lifespan extension 30. Furthermore, cells deleted in *SOV1* exhibit reduced PKA signaling and subsequent activation of the Msn2/4 stress response signaling. This results in improved cellular proteostasis and expression of Pnc1, which activates Sir2 via scavenging of nicotinamide, an inhibitor of Sir2‐mediated silencing (Fig 3, middle) 30, 76. Linking an additional feature of mitochondrial translation to lifespan control, we have recently shown that modulating the accuracy of mitochondrial translation by mutating the highly conserved accuracy center of mitochondrial ribosomes (Mrps12) determines cellular stress resistance, cytoplasmic proteostasis, and escape from senescence in yeast 17. Specifically, decreasing mitochondrial translation output boosts cytoplasmic protein folding and suppresses a Tor1‐dependent gene expression program that primarily targets genes implicated in mitochondrial proteostasis, leading to an extension of chronological lifespan. This novel interorganellar proteostasis transcription program also relies on the stress signaling transcription factors Msn2/4 and is induced during chronological aging to protect the cells from damage accumulation within mitochondria (Fig 3, bottom).

## Transcriptional responses to metabolic stress

Of importance to proteostasis and stress adaptation, cells have to adjust gene expression to the availability of energy sources to maintain optimal fluxes through fermentation and respiration. In yeast, these fluxes are principally determined by the availability of fermentable sugars. During growth in the presence of such preferred carbon sources, cellular metabolism displays resemblance to the Warburg effect in cancers and derives ATP predominantly from high rates of glycolysis followed by cytosolic fermentation to ethanol. Under scarcer nutritional conditions of glucose limitation, the cells exploit other carbon sources, upregulate mitochondrial activity, and rely on mitochondrial respiration for ATP generation. These cellular energy fluxes are intrinsically linked to proteostasis and stress conditions. First, the proteostasis system is fed unstructured proteins from translation, a process that is tightly controlled by energy availability and growth rate 77. Second, as outlined above, mitochondrial proliferation and thus the flux of newly translated proteins that are imported into the organelle directly impact on the flow through the proteostasis system. Finally, many stress conditions, including aging, compromise metabolism. This amalgamation of proteostasis and metabolism necessitates overlapping genetic targets for proteotoxic stress and metabolic transcriptional pathways.

### The general stress response: PKA, Msn2, and Msn4

**Figure 4:**
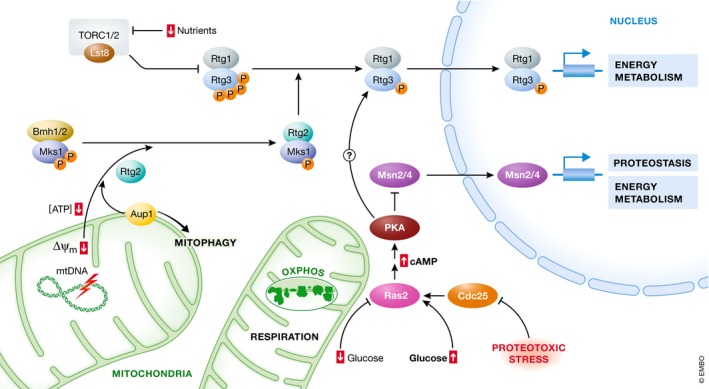
Transcriptional responses to metabolic stress Cells adjust gene expression to the availability of energy sources to maintain optimal fluxes through fermentation and respiration. These metabolic transcriptional pathways have overlapping genetic targets with pathways responding to proteotoxic stress. Top: The retrograde response is activated upon compromised mitochondrial energy metabolism. Binding of Mks1 to Rtg2 allows the heterodimeric transcription factors Rtg1 and Rtg3 to escape latency control and to translocate to the nucleus to activate the retrograde response genes. In addition, TORC1 signaling as well as Ras2/PKA signaling impacts on the retrograde response. Left: The phosphatase Aup1, a regulator of mitophagy, is required for an efficient induction of the retrograde response in aged cells, connecting mitophagic protein quality control to the retrograde response‐induced transcriptional reprogramming. Bottom: The PKA‐Msn2/4 regulon integrates metabolic and proteostatic cues into a broad transcriptional program that enables cells to adapt to starvation and stress.

In yeast, PKA controls cell growth, metabolic fluxes, and gene expression in response to carbon source availability 78. PKA‐dependent transcriptional regulation is executed by the downstream transcription factors Msn2/4, which are active under respiration and the key determinants and dominant effectors for the low‐glucose phenotype (Fig 4, bottom) 79. Activating Msn2 by blocking multiple PKA phosphorylation sites is sufficient to induce a shift of the transcriptional profile and metabolism toward glucose exhaustion 80. Msn2/4 play a dual role in activating metabolic and stress response genes and have been characterized as general stress factors 81, 82. Nevertheless, the mechanistic understanding of how Msn2/4 are activated in response to various stress regimes is rather limited, but likely involves low PKA activity. This is corroborated by the finding that Msn2/4 is dephosphorylated under PKA‐dependent stress regimes like glucose depletion or exposure to hydrogen peroxide 83, 84, 85. Such stress‐induced low PKA activity could be the result of perturbed glucose uptake, which is the rate‐limiting step in sugar metabolism and hence controls PKA activity 86. Alternatively, stress could impact on the upstream cAMP signaling module. This module involves Cdc25, the guanine nucleotide exchange factor for Ras2, which in turn activates the adenylate cyclase. Cdc25 has been suggested to function as a sensor and transducer of stress 87. Furthermore, Cdc25 represents a putative factor for the modulation of the cAMP pathway by the proteostasis system. Its function is positively regulated by cytosolic Hsp70, suggesting that proteostasis perturbations titrate the chaperone away from Cdc25 88, 89. This results in decreased Cdc25 activity and reduced cAMP production, in consequence inhibiting PKA‐dependent transcription. In sum, PKA signaling integrates metabolic and stress cues into a broad transcriptional program that enables cells to adapt to starvation conditions and stress.

Transcriptional reprogramming via Msn2/4 is critical for cells to undergo metabolic transition during the diauxic shift, i.e., the change upon exhaustion of glucose from fast fermentative growth to mitochondrial respiratory growth (Fig 4). Due to its prominent role in the diauxic shift, execution of this program is a prerequisite for viability during chronological aging 90, 91, 92. Specifically, Msn2/4 function as the downstream effector of interorganellar proteostasis signals. For instance, accuracy of mitochondrial translation during aging regulates Msn2/4‐dependent IPTP, which induces genes important for mitochondrial biogenesis and proteostasis 17. How this signaling is wired to connect mitochondria to the nucleus and whether it involves altered PKA activity remain unknown. Curiously, accuracy of mitochondrial translation directly impacts on cytosolic proteostasis (Fig 3, bottom), suggesting that this signaling could rely on similar mechanisms as other proteostatic stressors to feed information into the PKA‐Msn2/4 regulon. Hence, manipulating the accuracy of mitochondrial translation and consequently proteostasis has profound effects on Msn2/4‐dependent control of lifespan, linking proteostatic and metabolic mitochondria‐to‐nucleus signaling pathways.

### Metabolic adaptation via the retrograde response

Distinct mitochondrial stress regimes that compromise mitochondrial energy metabolism trigger transcriptional reprogramming via the retrograde response (Fig 4) 31, 32, 33. The activation of the retrograde response fosters energy metabolism when respiration fails and contributes to both replicative and chronological longevity 15, 93, 94, 95. While a causal link between aging and loss of mitochondrial transmembrane potential has been questioned 96, it is generally assumed that the progressive decrease of mitochondrial transmembrane potential in aging cells is accompanied by an activation of the retrograde response 97, 98. The key nuclear effectors of the retrograde pathway are the heterodimeric transcription factors Rtg1 and Rtg3 (Rtg1/3), which are controlled by the upstream components Rtg2 and the sequestering factor Mks1 (Fig 4, top) 31. Mks1 is a negative regulator of the retrograde response that maintains Rtg3 phosphorylated and prevents the nuclear translocation of the heterodimeric Rtg1/3 complex. Activation of this pathway involves inactivation of Mks1 by binding to Rtg2. This allows Rtg1/3 to escape latency control and enter the nucleus to activate retrograde response genes 99. Several additional regulatory factors have been identified, including Lst8, an essential factor in Tor1 signaling, connecting the retrograde response to this major stress signaling pathway 31, 100. Furthermore, inhibition of TORC1 signaling via nitrogen starvation or rapamycin treatment promotes longevity and activates the retrograde response 101, 102. In addition, inactivation of Ras2/PKA signaling abrogates retrograde response‐dependent transcriptional changes, thus impeding longevity of cells lacking mitochondrial DNA (Fig 4, bottom) 15.

Interestingly, mitophagy, the selective autophagic degradation of mitochondria, has been linked to regulators of the retrograde response. In chronologically aged cells, an efficient retrograde response requires the mitochondrial intermembrane space protein Aup1, a phosphatase that governs mitophagy (Fig 4, left) 94. This connects the quality control exerted by mitophagy to compensatory mitochondria‐to‐nucleus communication. Mechanistically, increased expression of retrograde response‐target genes precedes the induction of mitophagy 94. This indicates that this transcriptional reprogramming not only switches cellular metabolism but also primes the cell for efficient degradation of dysfunctional mitochondria.

Mitochondrial energy fluxes are fundamentally linked to the induction of retrograde response. A candidate signaling molecule is ATP that at physiological levels negatively controls the critical regulatory step of the retrograde response, which is the interaction between Mks1 and the ATP‐binding protein Rtg2 103. Accordingly, decreased ATP levels activate the retrograde response by allowing Rtg2 to bind Mks1. While the induction of the retrograde response has mostly been studied in cells lacking mitochondrial DNA, dissipation of mitochondrial transmembrane potential is sufficient to activate the retrograde response and extend replicative lifespan 104. However, a decrease in transmembrane potential has direct consequences for mitochondrial energy conversion and cellular ATP levels. In addition, and as previously discussed, suboptimal protein import into mitochondria, as provoked by reduced mitochondrial transmembrane potential, represents a cue that may be sensed in the cytosol and thus trigger the retrograde response.

## Concluding remarks

Mitochondria function as hubs that integrate metabolic and proteostatic fluxes to modulate stress responses to various insults. The so far described mechanisms connecting mitochondria and the rest of the cell illustrate the complexity of wiring between the systems. Instead of specific gene‐regulatory receptors responding to distinct cues, cells appear to monitor metabolic and proteostasis fluxes and feed information into general proteostatic and metabolic transcriptional programs. The theme is conserved across species barriers. For instance, the canonical mammalian UPR^mt^ is triggered by protein misfolding in mitochondria but depends on general stress factors and highly complex promoters to change the nuclear gene expression of mitochondrial chaperones 105. In yeast, decreased chaperone function in the cytosol, mitochondria, and the ER results in decreased respiration, metabolic changes, and activation of gene‐regulatory programs that fortify proteostasis in the cytosol, ER, and mitochondria 106.

The limited current mechanistic understanding raises the question if the complex interplay of stress responses and mitochondria can be conceptually approached. An emerging explanation for the existence of so many overlapping general stress response programs is that they are monitoring damage to shared processes and molecular targets. Presumably, flows through metabolic and proteostatic pathways represent these common targets for stress‐induced damage. Accumulation of unfolded proteins represents a burden on the proteostasis system, which is alleviated by the organellar import of these polypeptides. Stress impedes organellar protein import, resulting in titration of cytosolic chaperones, including Hsp70. This allows to regulate the activity of transcription factors that are associated with chaperones such as Hsf1 and Pdr3. Organellar protein import is itself a chaperone‐driven mechanism and therefore highly dependent on efficient proteostatic flow between the organelles and the cytosol. Importantly, mitochondria generate ATP, the fuel of chaperone‐dependent protein folding and organellar import. Accordingly, ATP levels are a common metabolic cue for stress signaling. In addition to the chaperone‐titrated mechanisms that sense ATP levels indirectly via mitochondrial protein import, the classical retrograde response is initiated by an ATP‐regulated step and Msn2/4 depends on metabolic PKA signaling. Thus, the mitochondrion is an ideal organelle to monitor and integrate proteostasis and energy cues with the output of transcriptional regulation.

The findings regarding how mitochondria orchestrate proteostatic and metabolic stress responses in the yeast model have implication for the understanding of this wiring also in other eukaryotes, including humans and pathological conditions. In the rapidly evolving regulatory networks, the molecular details of the wiring are predicted to differ between organisms, yet the basic concepts may remain. For example, the UPRmt in *C. elegans* involves the transcription factor ATFS‐1 that is not present in yeast or humans but still relies on tapping into the information provided by the functionality of the mitochondrial protein import system. Clinically relevant mitochondrial damage is known to result in mitochondrial precursor over‐accumulation stress, so‐called mPOS that is characterized by aberrant accumulation of mitochondrial precursors in the cytosol and is suppressed by protein biogenesis genes 107. Again on the conceptual level, ongoing mitochondrial translation signals to the cytosolic proteostasis system in yeast and similarly is required to induce a stress response in mammals, yet the molecular wiring will likely differ 108. On the contrary, examples of conserved components also exist. The ancient TOR signaling pathway is involved in mitochondrial signaling not only in yeast but mTORC1 is also required for the integrated mitochondrial stress response called ISRmt that controls metabolic transcription, one‐carbon metabolism, and the mitoUPR in mitochondrial myopathy 109. The key concept emerging from the study of the yeast model and of value for other eukaryotic systems is thus that future research efforts should be directed toward understanding the fluxes of unfolded proteins and metabolites within the interconnected proteostasis and metabolic systems.

### Conflict of interest

The authors declare that they have no conflict of interest.

**In need of answers**

Despite the elucidation of many basic aspects of mitochondria‐to nucleus signaling in recent years, some central questions remain unanswered.

- What are the signaling molecules for mitochondria‐nuclear crosstalk? Relative ATP levels have been suggested to play an important role, but how ATP derived from respiration can be differentiated from ATP produced during glycolysis is unclear. Are other, more mitochondria‐specific metabolites utilized in this signaling?
- How are mitochondrial and cytoplasmic proteostasis wired? While methods have been established to determine the state of cytoplasmic proteostasis, assays allowing to follow protein (re‐)folding and the dynamics of aggregate handling in mitochondria need to be developed.
- How are different signaling routes coordinated and are there specific nodes for signal integration? In light of the plethora of mitochondrial‐to‐nucleus signaling pathways, it is likely that they all impinge on central routes, but provoking different transcriptional responses by modulation of transcription factors.
- How can mitochondrial translation, which produces primarily hydrophobic membrane proteins, impact cellular stress by routes distinct of energy metabolism?
